# Plasma Levels of Alanine Aminotransferase in the First Trimester Identify High Risk Chinese Women for Gestational Diabetes

**DOI:** 10.1038/srep27291

**Published:** 2016-06-06

**Authors:** Junhong Leng, Cuiping Zhang, Peng Wang, Nan Li, Weiqin Li, Huikun Liu, Shuang Zhang, Gang Hu, Zhijie Yu, Ronald CW Ma, Juliana CN Chan, Xilin Yang

**Affiliations:** 1 Tianjin Women and Children’s Health Centre, Tianjin, China; 2 Department of Epidemiology and Biostatistics, School of Public Health, Tianjin Medical University, Tianjin, China; 3 Chronic Disease Epidemiology Laboratory, Pennington Biomedical Research Center, Baton Rouge, Louisiana, USA; 4 Population Cancer Research Program and Department of Pediatrics, Dalhousie University, Halifax, Canada; 5 Department of Medicine and Therapeutics, Hong Kong Institute of Diabetes and Obesity and The Chinese University of Hong Kong, Hong Kong SAR, China

## Abstract

Alanine aminotransferase (ALT) predicts type 2 diabetes but it is uncertain whether it also predicts gestational diabetes mellitus (GDM). We recruited 17359 Chinese women with ALT measured in their first trimester. At 24–28 weeks of gestation, all women underwent a 50-gram 1-hour glucose challenge test (GCT) followed by a 75-gram 2-hour oral glucose tolerance test if GCT result was ≥7.8 mmol/L. Restricted cubic spline analysis was used to examine full-range risk associations of ALT levels with GDM. Relative excess risk due to interaction, attributable proportion due to interaction and synergy index were used to estimate additive interaction between high ALT and overweight/obesity for GDM. Finally, 1332 (7.7%) women had GDM. ALT levels were positively associated with GDM risk without a clear threshold. Using ALT levels <22 U/L as the referent, the middle ALT levels (≥22 to <40 U/L) [odds ratio (OR) (95% confidence intervals): 1.41(1.21–1.65)] and high ALT levels (≥40 U/L) [1.62 (1.31–2.00)] were associated with increased GDM risk. Maternal overweight/obesity greatly enhanced the OR of ALT ≥22 U/L from 1.44 (1.23–1.69) to 3.46 (2.79–4.29) with significant additive interactions. In conclusion, elevated ALT levels in the first trimester even within normal range predicted GDM risk, further enhanced by overweight/obesity.

Gestational diabetes mellitus (GDM) is defined as any degree of glucose intolerance with onset or first recognition during pregnancy^1^. GDM is associated with adverse short-term and long-term health outcomes in both mothers and their offspring^2^,^3^. The prevalence of GDM has been increasing worldwide^4^. In Tianjin, China, the prevalence of GDM increased from 2.3% in 1999 to 9.3% in 2010–2012^5^. Although our group^6^ and others^7^ showed that lifestyle intervention was able to improve pregnancy outcomes, a recent trial failed to show that lifestyle intervention was able to reduce GDM risk^8^. Since GDM is likely to be caused by multiple factors, it is pivotal to ascertain novel risk factors and study interactions amongst risk factors in order to identify high risk women for intervention during or before pregnancy.

Liver plays an important role in glucose metabolism. Hepatocytic enzymes, namely, alanine aminotransferase (ALT), g-glutamyl transferase (GGT) and aspartate aminotransferase are associated with insulin resistance^9^,^10^ and type 2 diabetes^11^,^12^,^13^,^14^. Amongst them, ALT is primarily aggregated in the liver and serves as a more-specific and better marker for liver fat deposition and non-alcoholic fatty liver disease (NAFLD). NAFLD is characterized by elevated hepatic fat content which causes hepatic insulin resistance to increase the risk of type 2 diabetes^15^. Since GDM and type 2 diabetes share many common risk factors and similar pathogenesis, it is plausible that elevated ALT levels may also predict GDM, although such data are relatively scarce and inconsistent. In a survey where ALT was measured two weeks before performance of oral glucose tolerance test (OGTT)^16^, the authors did not find association between ALT levels and GDM risk. Maternal overweight and obesity was a well-recognized predictor for GDM^17^. In northern Taiwan, people with co-occurrence of elevated ALT and abdominal obesity had a synergistic effect on the risk of incident diabetes mellitus^18^. With the increasing prevalence of obesity worldwide, we hypothesize that overweight/obesity and elevated ALT levels may have synergistic effect on GDM risk.

In this prospective cohort of pregnant women established from 2010 to 2012 in Tianjin, China, we aimed to examine 1) whether ALT levels in the first trimester predicted GDM in Chinese women; and 2) whether elevated ALT and maternal overweight/obesity in the first trimester had a synergistic effect towards increasing GDM risk.

## Methods

### Study population and settings

Tianjin is a metropolitan city in northern China, covering an area of 11,900 square kilometers, with six central urban districts, one new urban district, four suburban districts and five counties. The city has a population of over 13 million and about 4.3 million of them live in the six central urban districts. The detailed description of the methods and characteristics of the cohort had been described^5^. Briefly, from October 2010 to August 2012, we set up a large cohort of pregnant women and their infants in the six central urban districts of Tianjin and documented clinical and biochemical profiles longitudinally from their first antenatal visit to delivery. During this period, 19 669 pregnant women aged ≥18 years registered for pregnancy at a primary care hospital within the first 12 weeks of gestation (range: 4–12 weeks) and received their first antenatal care there. All women underwent a two-step GDM screening procedure later in 24 to 28 weeks of gestation, using the 50-gram glucose challenge test (GCT). Those women with a screen positive threshold set at ≥7.8mmol/L were referred to a central GDM clinic located within Tianjin Women and Children’s Health Centre (TWCHC) to receive a diagnostic 75-gram OGTT. We sequentially excluded 1080 women who did not have GCT, 310 women with positive hepatitis B surface antigen (HBsAg), 156 women with missing ALT values and 764 women who had a positive GCT but did not undergo OGTT. Eventually 17 359 pregnant women were included in the analysis.The Ethics Committee for Clinical Research of TWCHC approved the study and each participant provided informed written consent. The methods were carried out in accordance with the Declaration of Helsinki.

### Screening for and diagnosis of GDM

All women underwent a 50-gram 1-hour GCT between 24 and 28 weeks of gestation at primary care hospitals. Plasma glucose (PG) levels were measured using non-fasting venous blood taken 60 minutes after the ingestion of 200 ml of 25% glucose solution. Women with positive results (PG ≥7.8mmol/L) were referred to the clinic of TWCHC to undergo a 75-gram 2-hour OGTT to confirm the diagnosis of GDM. Plasma glucose was measured three times (in the morning after overnight fasting of at least 8 hours, 1 hour and 2 hours after ingesting 300 ml of 25% glucose solution) using an automatic analyzer (Toshiba TBA-120FR, Japan) with a coefficient of variance <2.59% .The diagnosis of GDM was based on the International Association of Diabetes and Pregnancy Study Group (IADPSG) cut-points defined as fasting plasma glucose (FPG) ≥5.1 mmol/L or 1-hour PG ≥10.0 mmol/L or 2-hour PG ≥8.5 mmol/L^19^.

### Clinical and laboratory measurements and data collection

Serum ALT and HBsAg were measured after an overnight fast in conjunction with other routine antenatal blood investigations during the first antenatal care visit. ALT levels were tested using an automated enzymatic method (Toshiba TBA-120FR, Japan). The intra-assay and inter-assay coefficients of variation for ALT were 3.83% and 9.21% respectively. HBsAg was tested using enzyme-linked immunosorbent assay (ELISA) (Xiamen Xinchuang Assay Kit). No other liver function indicators were measured.

Maternal height, weight and blood pressure (BP) were measured using a standardized protocol by uniformly-trained nurses at the primary care hospitals during first antenatal care visit. Weight was measured to an accuracy of 0. 1 kg and height was measured to the nearest 0.5 cm. Weight were re-measured when GCT was performed. Because weight gain in the first trimester of pregnancy is small and negligible^20^, we used body weight at first antenatal care visit as pre-pregnancy body weight to estimate pre-pregnancy body mass index (BMI). BMI was calculated as weight at first antenatal care visit in kilogram (kg) divided by squared body height in meter (m). BMI groups at first antenatal care visit were defined based on the criteria recommended by World Health Organization (WHO): underweight (<18.5 kg/m^2^), normal weight (18.5 to 24.9 kg/m^2^), overweight (25.0 to 29.9 kg/m^2^) and obesity (≥30.0 kg/m^2^)^21^. Sitting BP was measured after at least 10 minutes of rest with a calibrated mercury sphygmomanometer.

Other data were longitudinally collected using a series of questionnaires completed by care providers/pregnant women at first antenatal care visit and GCT time, including maternal age, family history of diabetes in first degree relatives, parity, ethnicity, education attainment, habitual smoking before or during pregnancy, and alcohol consumption before or during pregnancy. Education attainment was classified into >12 years and ≤12 years of schooling. Habitual smoking before or during pregnancy was defined as continuously smoking one or more cigarettes per day for at least 6 months before pregnancy or smoking one or more cigarettes per day during pregnancy.

### Statistical analyses

Data were analyzed using IBM SPSS Statistics 20.0 (IBM SPSS, Chicago, IL), unless otherwise specified. A P value of <0.05 (two-tailed) was considered to be statistically significant. Due to the lack of practical guideline for abnormal ALT levels in the first trimester of pregnancy and considering that the association between ALT levels and GDM risk might be nonlinear, restricted cubic spline (RCS) analysis was used in univariable and multivariable binary logistic regression models to derive OR curves to capture full range associations between ALT and GDM risk, which was used to guide to stratify ALT in subsequent analysis. The Statistical Analysis System (release 9.10) was used to perform the RCS analysis (SAS Institute, Cary, NC). The RCS analysis uses piecewise cubic polynomials that are connected across different intervals of a continuous variable. It can fit sharply curving shapes, with the additional advantage that only k–1 parameters must be estimated (where k is the number of knots)^22^. We chose 5 knots at quantiles 0.050, 0.275, 0.500, 0.725 and 0.950, since it has been suggested that 4 or 5 knots offer an adequate fit of the model, representing a good compromise between flexibility and the loss of precision caused by overfitting a small sample. Use of the RCS in binary regression models has been described in detail by Harrell^22^. Similar to our previous analyses^23^, the OR of points 2 versus 1 of variable X can be estimated by exp (Y2–Y1), where Y1 and Y2 are the corresponding spline function values of the 2 points of variable X. If we select a proper point Y1 as the referent, exp (Y2–Y1) stands for the OR of point 2 versus point. In this study, the value of ALT with the lowest GDM risk was used as the referent, and the ORs of all other ALT levels versus the referent value were calculated and plotted against their respective ALT levels. Because there were no clear-cut threshold points of ALT for GDM, we arbitrarily stratify ALT into three groups at two cutoff points, 22 U/L (also 75^th^ percentile) and 40 U/L, i.e., the recommended cutoff point to define abnormal liver function^24^.

To compare the difference between GDM group and non-GDM group, Student t-test or Mann-Whitney U-test was used for continuous variables, and Chi-square test or Fisher’s exact test was used for categorical variables. Binary logistic regression models were used to obtain odds ratios (ORs) and their 95% confidence intervals (CIs) in univariable and multivariable analysis. Multivariable analysis was adjusted for maternal age, height, family history of diabetes, parity, education, Han-ethnicity, gestational age at first antenatal care visit, systolic BP and BMI at first antenatal care visit, multiple pregnancies, habitual smoker before/during pregnancy, alcohol consumption before/during pregnancy, gestational age at GCT and weight gain from first antenatal care visit to GCT time .

The additive interaction between maternal overweight/obesity at first antenatal care visit and ALT levels for GDM risk was used to check possible synergistic effects. Three indicators, relative excess risk due to interaction (RERI), attributable proportion due to interaction (AP), and synergy index (S), were used to judge whether the additive interaction was statistically significant. A detailed calculation method was available (available at http://www.epinet.se)^25^. Briefly, RERI is the excess risk due to interaction relative to the risk without exposure. AP is the attributable proportion of disease that is due to interaction among individuals with both exposures. S is the excess risk from both exposures when there is an additive interaction, relative to the risk from both exposures without interaction. Significant RERI > 0, AP > 0 or S > 1 indicated an additive interaction.

Two sensitivity analyses were performed in independent and additive interaction models to check the impacts of exclusion of 764 women with positive GCT result but did not undergo OGTT. One was re-inclusion of these 764 women assuming that all had GDM and the other, assuming all did not have GDM. In all analyses, P values and 95% CI derived from multiple comparisons were adjusted using the Ryan–Holm step-down Bonferroni procedure^26^.

## Results

### Characteristics of the study population

Amongst 17 359 women who attended the first antenatal care visit, mean age was 28.5 (SD: 2.8) years, mean height was 163.2 (SD: 4.7) cm, mean BMI at first antenatal care visit was 22.3 (SD: 3.4) kg/m^2^, and mean diastolic/systolic BP at first antenatal care visit were 68.4/105.6 (SD: 7.7/10.6) mmHg. Of all women, 96.8% were nulliparous, 15.6% were overweight and 3.0% were obese. The median ALT levels at first antenatal care visit was 15 U/L (Interquartile range: 11–22 U/L). A total of 1332 women (7.7%) were diagnosed to have GDM. The women with GDM were older, shorter, had higher BMI and systolic/diastolic BP at first antenatal care visit, were more likely to be multipara and Han-ethnicity, have a family history of diabetes in first degree relatives and habitual use of tobacco before pregnancy than women without GDM (Table 1). The women with GDM had higher median ALT levels at first antenatal care visit (18 U/L vs. 15 U/L, P <0.001).

### The risk associations of ALT levels and maternal overweight/obesity with GDM

**Figure 1:**
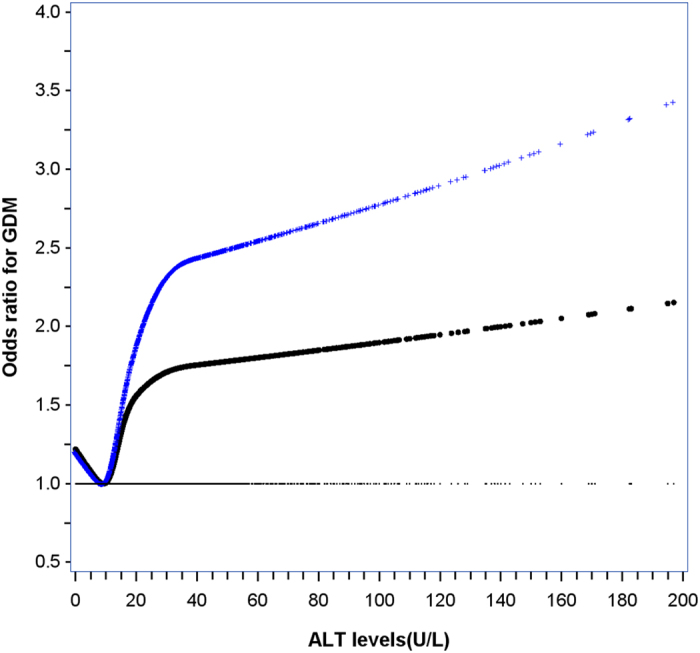
Full range associations between alanine aminotransferase (ALT) in first trimester and the risk of gestational diabetes mellitus (GDM) in Chinese pregnant women. The cross (upper, blue) line is the unadjusted curve and the dotted (bottom, black) line is the adjusted curve. The adjusted curve was derived from multivariable logistic regression with adjusting for maternal age, height, family history of diabetes, parity, education, Han-ethnicity, gestational age at first antenatal care visit, BMI and systolic blood pressure at first antenatal care visit, multiple pregnancies, habitual smoker before/during pregnancy, alcohol consumption before/during pregnancy, weight gain from first antenatal care visit to the time of glucose challenge test and gestational age at the time of glucose challenge test (P<0.001).

On spline analysis, the ALT level was linearly associated with OR of GDM starting at level well below 22 U/L and rapidly accelerated reaching a plateau of 40 U/L (5.6% of women were ≥40 U/L) (Figure 1). The effect size was attenuated after adjusting for covariables but remained significant. In univariable analysis, both elevated ALT levels and maternal overweight/obesity at first antenatal care visit were associated with increased risk of GDM (Table 2). In multivariable analysis, after adjustment for covariates, compared to the reference group (ALT<22 U/L), middle (22 to 40 U/L) [adjusted OR: 1.41 (1.21–1.65)] and high levels (≥40 U/L) [adjusted OR: 1.62 (1.31–2.00)] of ALT were associated with GDM in a graded manner (P value for trend: <0.001). Compared to normal weight at first antenatal care visit (BMI: 18.5 to 24.9 kg/m^2^), overweight [adjusted OR: 2.10 (1.80–2.46)] and obesity [adjusted OR: 2.68 (1.96–3.65)] were also associated with GDM in a graded manner (P value for trend : <0.001).

Additional analysis was performed with further adjustment for PG levels at GCT time. Compared to the reference group (ALT<22 U/L), ALT middle levels (22 to 40 U/L) was still associated with GDM [adjusted OR: 1.22 (1.01–1.46)] but ALT high levels (≥40 U/L) was not associated with GDM [adjusted OR: 1.11 (0.83–1.49)].

### Additive interaction between ALT levels and maternal overweight/obesity for GDM risk

Using the ALT level <22 U/L and BMI <25 kg/m^2^ as the referent group, maternal overweight/obesity at first antenatal care visit increased the adjusted OR of elevated ALT levels (≥22 U/L) for GDM risk from 1.44 (1.23 to 1.69) to 3.46 (2.79 to 4.29) with significant additive interaction (P < 0.05 for RERI, AP and S) (Tables 2 and 3). Similarly, elevated ALT levels (≥22 U/L) increased the adjusted OR of maternal overweight/obesity at first antenatal care visit for GDM risk from 2.22 (1.84 to 2.68) to 3.46 (2.79 to 4.29) with significant additive interaction (P < 0.05 for RERI, AP and S).

### Sensitivity analyses

We re-included the 764 women who did not undergo OGTT and assumed all of them did not have GDM. In a similar multivariable model, compared to ALT referent group (<22 U/L), the OR of middle level was 1.39 (1.19–1.62) and that of high level group was 1.55 (1.26–1.92) for GDM risk (Table 4). If we assumed all women to have GDM, the respective ORs were 1.41 (1.26–1.58) and 1.67 (1.37–2.04). The two sensitivity analyses of additive interaction models showed a consistent trend towards an interactive effect between elevated ALT levels and maternal overweight/obesity on GDM risk, reaching significance for both models (Table 5).

## Discussion

In Chinese pregnant women, high ALT levels in the first trimester even within normal range were independently associated with increased risk of GDM, without an apparent threshold. In addition, elevated ALT levels arbitrarily defined as ≥22 U/L and maternal overweight/obesity defined as ≥25 kg/m^2^ had a synergistic effect towards increasing the risk of GDM.

There are consistent epidemiological data showing the associations between raised ALT levels and increased risk of type 2 diabetes^11^,^12^,^13^,^14^. To date, only two studies investigated risk associations between ALT levels and GDM and both had negative findings^16^,^27^. The discrepancy between findings of the two studies and ours is likely due to differences in study designs, sample size, populations, timing of ALT measurement, screening procedures and diagnostic criteria for GDM. The first negative report involved a nested case-control study, including 256 women with GDM selected from a cohort of 4098 women who participated in the Kaiser Permanente Northern California (KPNC) multiphasic health checkup from 1984 to1996 and had a subsequent pregnancy from 1984 to 2009^27^. The ALT level, on average, 7 years prior to index pregnancy, was used to examine the risk association between pre-pregnancy ALT levels and incident GDM. The long time gap may be one of the reasons for the lack of association between ALT and GDM since many modifying factors including lifestyle might have occurred during this interim period^27^. The other was a cross-sectional study from Malaysia which included 2610 pregnant women with 319 cases of GDM^16^ and the negative result might be due to insufficient power. Besides, the ALT level during pregnancy was, on average, measured two weeks prior to OGTT. The authors also recognized that the relatively short time gap could not have “predictive” values given that chronic elevation of ALT would be a more appropriate risk marker of GDM.

The association between elevated ALT and GDM risk may be due to shared pathophysiological mechanisms between GDM and type 2 diabetes as both diseases share many common risk factors. ALT is a cytosolic enzyme which has the highest concentrations in the liver. In the presence of liver injury, such as infection, toxins and ischemia, ALT is released from injured liver cells with elevated serum ALT. On the other hand, mild elevation of ALT level, often within normal range may reflect fat accumulation, a marker of NAFLD, rather than liver injury^28^. NAFLD is strongly associated with obesity and insulin resistance^13^,^29^ which lead to type 2 diabetes, in part mediated through endothelial dysfunction^30^,^31^, especially in subjects with other predisposing factors such as genetics.

Diabetes and hyperglycemia are a complex metabolic condition due to multiple causes. Apart from age, gender, family history, genetic and socioeconomic factors , overweight and obesity is an important modifiable risk factor for GDM^17^, which is similar to type 2 diabetes with a rapidly increasing prevalence world-wide. In this study, elevated ALT and maternal overweight/obesity had a synergistic effect on GDM risk. A cross-sectional study from northern Taiwan involving 1308 male workers aged 22 to 63 years^18^, observed that obesity and elevated ALT had a synergistic effect on insulin resistance (S: 2.1, 95% CI: 1.01–4.3). In another study from middle Taiwan including 5499 subjects, co-occurrence of obesity and elevated ALT was associated with higher odds for diabetes risk compared to subjects with only one of the two risk factors^32^. Compared to the non-obese group with ALT ≤ 40 U/L, the obese and ALT ≤ 40 U/L (adjusted OR: 1.73, 95%CI: 1.08–2.77), the obese and ALT 41–80 U/L (adjusted OR: 2.06, 1.20–3.55) , the obese and ALT 81–120 U/L [adjusted OR: 3.07, 1.38–6.84) and the obese and ALT > 120 U/L (adjusted OR: 7.44, 3.04–18.18) were all associated with increased risks of diabetes^32^. These findings reiterate the importance of using multiple risk factors to identify high risk subjects. In the case of GDM, since these women are likely to undergo blood tests for various reasons, our results suggest that early measurement of ALT may help to identify women at high risk of GDM, especially in the presence of obesity/overweight. Indeed, according to Bacq *et al.*^33^, pregnant women in the first trimester had similar ALT levels as non-pregnant women. Thus, elevated ALT level during the first trimester could identify women at risk of developing GDM in later pregnancy so to make possible early detection and prevention.

Our study had strengths. First, our study was a large prospective population-based study with a low dropout rate (10.2%). Second, we had detailed documentation of traditional GDM risk factors and confounders for elevated ALT levels, e.g. use of alcohol and chronic HBV infection, which were excluded or adjusted in multivariable model. Third, ALT levels were measured in the first trimester, on average, about 15 weeks before identification of GDM later in pregnancy. Our study also had limitations. First, we excluded 746 women who had GCT but did not undergo OGTT. Exclusion of these high risk women may overestimate the risk for GDM while inclusion of these women with the assumption that they did not have GDM may underestimate the risk for GDM in the study population. However, our sensitivity analysis suggested that attenuations of the observed effect sizes were very small and therefore major biases were unlikely. Second, we were unable to examine GGT and other liver enzymes levels known to be associated with type 2 diabetes^11^,^12^. Their predictive values for GDM need further investigations. Third, detailed lifestyle factors such as dietary habits and physical activity were not available for adjustment. Fourth, some women with GDM might have been missed using the two-step procedure although this protocol has been used since 1999 to minimize logistic problems. Misclassification of GDM as non GDM is more likely to lead to underestimation of the effect size. Hence, the true effect size of ALT levels on GDM might be higher than what this study reported.

In conclusion, elevated ALT in the first trimester was associated with increased GDM risk without a clear threshold and maternal overweight/obesity at first antenatal care visit further enhanced the risk association between high ALT and GDM. In addition to documenting conventional risk factors including overweight/obesity, measurement of ALT levels in the first trimester can be used to identify high risk women for early detection and prevention of GDM if these findings can be replicated in other cohorts.

## Additional Information

**How to cite this article**: Leng, J. *et al.* Plasma Levels of Alanine Aminotransferase in the First Trimester Identify High Risk Chinese Women for Gestational Diabetes. *Sci. Rep.* **6**, 27291; doi: 10.1038/srep27291 (2016).

## Figures and Tables

**Table 1:** Clinical and biochemical characteristics of subjects according to occurrence of gestational diabetes mellitus.

	Non GDM	GDM	P-value
N	16027	1332	
Variables at first antenatal care visit			
Age, year	28.4 ± 2.8	29.5 ± 3.1	<0.001*
Age group, year			<0.001**
<30	12275(76.6%)	871(65.4%)	
≥30–<35	3367(21.0%)	385(28.9%)	
≥35	385(2.4%)	76(5.7%)	
Height, cm	163.2 ± 4.7	162.7 ± 4.7	<0.001*
BMI, kg/m^2^	22.1 ± 3.3	24.1 ± 3.9	<0.001*
BMI group, kg/m^2^			<0.001**
<18.5	1676(10.5%)	47(3.5%)	
≥18.5–<25	11607(72.4%)	799(60.0%)	
≥25–<30	2318(14.5%)	388(29.1%)	
≥30	420(2.6%)	98(7.4%)	
Gestational age at first antenatal care visit, weeks	9.8 ± 1.6	9.8 ± 1.6	0.849*
Diastolic blood pressure, mmHg	68.3 ± 7.7	70.4 ± 8.0	<0.001*
Systolic blood pressure, mmHg	105.3 ± 10.5	108.4 ± 11.0	<0.001*
Parity≥1	500(3.1%)	53(4.0%)	0.086**
Han-ethnicity	15297(95.4%)	1288(96.7%)	0.033**
Family history of diabetes in first degree relatives	1208(7.5%)	184(13.8%)	<0.001**
Education >12 years	13475(84.2%)	1099(82.7%)	0.148**
Multiple pregnancies	154(1.0%)	20(1.5%)	0.060**
ALT level, U/L	15(11–21)	18(13–26)	<0.001§
<22	12178(76.0%)	853(64.0%)	<0.001**
≥22–<40	3003(18.7%)	359(27.0%)	
≥40	846(5.3%)	120(9.0%)	
Variables at GCT			
Gestational age at GCT, weeks	24.8 ± 2.5	24.8 ± 1.8	0.091*
Weight gain from first antenatal care visit to GCT, kg	7.5 ± 3.4	7.5 ± 3.6	0.453*
Smoking habit			
Habitual smoker before pregnancy†	473(3.0%)	55(4.1%)	0.016**
Habitual smoker during pregnancy‡	98(0.6%)	11(0.8%)	0.341**
Alcohol drinking habit			
Alcohol drinker before pregnancy	4935(30.8%)	413(31.0%)	0.871**
Alcohol drinker during pregnancy	115(0.7%)	10(0.8%)	0.890**

Data are reported in mean ± SD or number (%) or median (interquartile range).

BMI, body mass index; GCT, glucose challenge test; ALT, alanine aminotransferase.

^*^Derived from Student’s t- test.

^**^Derived from Chi-square test or Fisher’s Exact Test.

^§^Derived from Mann-Whitney U- test.

^†^Defined as having continuously smoked one or more cigarette per day for at least six months.

^‡^Defined as having smoked one or more cigarette per day during pregnancy.

**Table 2:** Odds ratios (ORs) of alanine aminotransferase level for the risk of gestational diabetes mellitus.

	N (%)	OR	95%CI	P-value
Independent models				
Univariable analysis*				
ALT level, U/L				<0.001§
<22	853 (6.5%)	1.00	Reference	
≥22–<40	359 (10.7%)	1.71	1.50–1.94**	<0.001**
≥40	120 (12.4%)	2.03	1.60–2.56**	<0.001**
BMI group, kg/m^2^				<0.001§
<18.5	47 (2.7%)	0.41	0.30–0.55**	<0.001**
≥18.5–<25	799 (6.4%)	1.00	Reference	
≥25–<30	388 (14.3%)	2.43	2.10–2.82**	<0.001**
≥30	98 (18.9%)	3.39	2.56–4.50**	<0.001**
Multivariable analysis†				
ALT level, U/L				<0.001§
<22	853 (6.5%)	1.00	Reference	
≥22–<40	359 (10.7%)	1.41	1.21–1.65**	<0.001**
≥40	120 (12.4%)	1.62	1.31–2.00**	<0.001**
BMI group, kg/m^2^				<0.001§
<18.5	47 (2.7%)	0.49	0.36–0.66**	<0.001**
≥18.5–<25	799 (6.4%)	1.00	Reference	
≥25–<30	388 (14.3%)	2.10	1.80–2.46**	<0.001**
≥30	98 (18.9%)	2.68	1.96–3.65**	<0.001**
Additive interaction models				
Univariable analysis*				
ALT ≥22 U/L and BMI<25 kg/m^2^	247 (8.0%)	1.51	1.30–1.76**	<0.001**
ALT <22 U/L and BMI≥25 kg/m^2^	254 (12.8%)	2.55	2.14–3.05**	<0.001**
ALT ≥22 U/L and BMI≥25 kg/m^2^	232 (18.8%)	4.03	3.30–4.93**	<0.001**
ALT <22 U/L and BMI<25 kg/m^2^	599 (5.4%)	1.00	Reference	
Multivariable analysis‡				
ALT ≥22 U/L and BMI<25 kg/m^2^	247 (8.0%)	1.44	1.23–1.69**	<0.001**
ALT <22 U/L and BMI≥25 kg/m^2^	254 (12.8%)	2.22	1.84–2.68**	<0.001**
ALT ≥22 U/L and BMI≥25 kg/m^2^	232 (18.8%)	3.46	2.79–4.29**	<0.001**
ALT <22 U/L and BMI<25 kg/m^2^	599 (5.4%)	1.00	Reference	

N (%), number of cases (% of number at risk).

^*^not adjusted for any other variables.

^**^P values and 95%CIs of ORs were adjusted for multiple comparisons by Ryan-Holm step-down Bonferroni procedure.

^†^variables adjusted in the multivariable analysis included age, height, family history of diabetes, parity, education, Han-ethnicity, gestational age at first antenatal care visit, systolic blood pressure, multiple pregnancies, habitual smoker before/during pregnancy, alcohol drinker before/during pregnancy, gestational age at the time of GCT and weight gain from first antenatal care visit to GCT, in addition to the variables listed in the model.

^‡^variables adjusted in the multivariable analysis included age, height, family history of diabetes, parity, education, Han-ethnicity, gestational age at first antenatal care visit, systolic blood pressure, multiple pregnancies, habitual smoker before/during pregnancy, alcohol drinker before/during pregnancy, gestational age at the time of GCT and weight gain from first antenatal care visit to GCT, in addition to the variables listed in the model.

^§^P value for trend.

**Table 3:** Additive interaction between ALT level ≥22 U/L and BMI≥25 kg/m^2^ for the risk of gestational diabetes mellitus.

Measures of additive interaction	Estimate(95%CI)**	P value
Univariable analysis*		
RERI	0.967(0.272, 1.662)	0.006
AP	0.240(0.093, 0.387)	0.001
S	1.468(1.118, 1.929)	<0.001
Multivariable analysis‡		
RERI	0.799(0.173, 1.424)	0.012
AP	0.231(0.075, 0.386)	0.004
S	1.480(1.090, 2.010)	<0.001

^*^not adjusted for any other variables.

^‡^the variables adjusted are the same as those in Table 2.

^**^statistically significant with RERI > 0, AP > 0 or S > 1 indicating significant additive interaction.

**Table 4:** Multivariable sensitivity analysis of odds ratios (ORs) of alanine aminotransferase level for the risk of gestational diabetes mellitus.

	N (%)	OR	95%CI	P-value
*Sensitivity analysis one*				
Independent models^†^				
ALT level, U/L				<0.001§
<22	853 (6.3%)	1.00	Reference	
≥22–<40	359 (10.1%)	1.39	1.19–1.62*	<0.001*
≥40	120 (11.6%)	1.55	1.26–1.92*	<0.001*
BMI group, kg/m^2^				<0.001§
<18.5	47 (2.6%)	0.49	0.36–0.67*	<0.001*
≥18.5–<25	799 (6.2%)	1.00	Reference	
≥25–<30	388 (13.5%)	2.05	1.76–2.40*	<0.001*
≥30	98 (17.2%)	2.49	1.83–3.38*	<0.001*
Additive interaction model^‡^				
ALT ≥22 U/L and BMI<25kg/m^2^	247 (7.6%)	1.41	1.21–1.66*	<0.001*
ALT <22 U/L and BMI≥25 kg/m^2^	254 (12.0%)	2.16	1.79–2.61*	<0.001*
ALT ≥22 U/L and BMI≥25 kg/m^2^	232 (17.3%)	3.27	2.64–4.04*	<0.001*
ALT <22 U/L and BMI<25 kg/m^2^	599 (5.2%)	1.00	Reference	
*Sensitivity analysis two*				
Independent models^†^				
ALT level, U/L				<0.001§
<22	1349 (10.0%)	1.00	Reference	
≥22–<40	545 (15.4%)	1.41	1.26–1.58*	<0.001*
≥40	190 (18.3%)	1.67	1.37–2.04*	<0.001*
BMI group, kg/m^2^				<0.001§
<18.5	101 (5.7%)	0.65	0.53–0.81*	<0.001*
≥18.5–<25	1270 (9.9%)	1.00	Reference	
≥25–<30	564 (19.6%)	1.93	1.69–2.21*	<0.001*
≥30	149 (26.2%)	2.61	2.00–3.39*	<0.001*
Additive interaction model^‡^				
ALT ≥22 U/L and BMI<25 kg/m^2^	399 (12.3%)	1.46	1.28–1.66*	<0.001*
ALT <22 U/L and BMI≥25 kg/m^2^	377 (17.9%)	2.05	1.75–2.40*	<0.001*
ALT ≥22 U/L and BMI≥25 kg/m^2^	336 (25.1%)	3.17	2.64–3.80*	<0.001*
ALT22 U/L and BMI<25 kg/m^2^	972 (8.5%)	1.00	Reference	

^*^P values and 95%CIs of ORs were adjusted for multiple comparisons by Ryan-Holm step-down Bonferroni procedure.

† and ‡, the variables adjusted are the same as in Table 2.

Sensitivity analysis one: Re-inclusion of the 764 subjects who had a positive GCT result but did not had a standard OGTT and assuming that all the 764 subjects did not have GDM.

Sensitivity analysis two: Re-inclusion of the 764 subjects who had a positive GCT result but did not had a standard OGTT and assuming that all the 764 subjects had GDM.

**Table 5:** Multivariable sensitivity analysis of additive interaction measures between ALT level ≥22 U/L and BMI≥25 kg/m^2^ for the risk of gestational diabetes mellitus.

Measures of additive interaction	Estimate (95%CI)*	P value
Sensitivity analysis one‡		
RERI	0.693(0.100, 1.285)	0.022
AP	0.212(0.053, 0.371)	0.009
S	1.440(1.054, 1.967)	<0.001
Sensitivity analysis two‡		
RERI	0.664(0.167, 1.161)	0.009
AP	0.209(0.073, 0.346)	0.003
S	1.441(1.098, 1.891)	<0.001

Sensitivity analysis one: Re-inclusion of the 764 subjects who had a positive GCT result but did not had a standard OGTT and assuming that all the 764 subjects did not have GDM.

Sensitivity analysis two: Re-inclusion of the 764 subjects who had a positive GCT result but did not had a standard OGTT and assuming that all the 764 subjects had GDM.

^‡^the variables adjusted are the same as those in Table 2.

^*^statistically significant with RERI > 0, AP > 0 or S > 1 indicating significant additive interaction.
